# Toward a Mechanistic Understanding of Color Vision in Insects

**DOI:** 10.3389/fncir.2018.00016

**Published:** 2018-02-23

**Authors:** Bo-Mi Song, Chi-Hon Lee

**Affiliations:** Section on Neuronal Connectivity, Eunice Kennedy Shriver National Institute of Child Health and Human Development, National Institutes of Health, Bethesda, MD, United States

**Keywords:** color vision, color opponency, chromatic information processing, wavelength selectivity

## Abstract

Many visual animals exploit spectral information for seeking food and mates, for identifying preys and predators, and for navigation. Animals use chromatic information in two ways. “True color vision,” the ability to discriminate visual stimuli on the basis of their spectral content independent of brightness, is thought to play an important role in object identification. In contrast, “wavelength-specific behavior,” which is strongly dependent on brightness, often associates with foraging, navigation, and other species-specific needs. Among animals capable of chromatic vision, insects, with their diverse habitats, stereotyped behaviors, well-characterized anatomy and powerful genetic tools, are attractive systems for studying chromatic information processing. In this review, we first discuss insect photoreceptors and the relationship between their spectral sensitivity and animals’ color vision and ecology. Second, we review recent studies that dissect chromatic circuits and explore neural mechanisms of chromatic information processing. Finally, we review insect behaviors involving “true color vision” and “wavelength-specific behaviors,” especially in bees, butterflies, and flies. We include examples of high-order color vision, such as color contrast and constancy, which are shared by vertebrates. We focus on *Drosophila* studies that identified neuronal correlates of color vision and innate spectral preferences. We also discuss the electrophysiological studies in bees that reveal color encoding. Despite structural differences between insects’ and vertebrates’ visual systems, their chromatic vision appears to employ the same processing principles, such as color opponency, suggesting convergent solutions of neural computation to common problems.

## Introduction

Light spectrum holds rich information about the world. The spectrum of natural illumination changes with daily cycle and objects differ in spectral reflection or emission properties (Spitschan et al., 2016). Like many other animals, insects use chromatic information to find favorable habitat, to efficiently locate food sources (Giurfa et al., 1997; Spaethe et al., 2001) and to identify conspecific mates using the spectral information (Finkbeiner et al., 2014; Huang et al., 2014). Pollinating insects such as many species of bees and butterflies use their color vision to maximize success in foraging. They detect flowers, memorize the colors and patterns of rewarding flowers, and preferentially collect nectars from the flowers in their later visits (von Helverson, 1972). Color vision is also critical for increasing efficiency in reproduction (Finkbeiner et al., 2014; Huang et al., 2014). For the *Heliconius erato* butterflies, identifying conspecific males is the basis for both male territoriality and female mate-choice (Bernard and Remington, 1991; Finkbeiner et al., 2014). Altering wing coloration changed the behaviors in both sexes substantially, suggesting that the butterflies use color vision for identifying conspecific males. Damselflies also appear to use color vision for mate choice. Body coloration of female butterflies changes with sexual maturation and aging and a strong correlation between female body color and mating was found (Huang et al., 2014).

**FIGURE 1:**
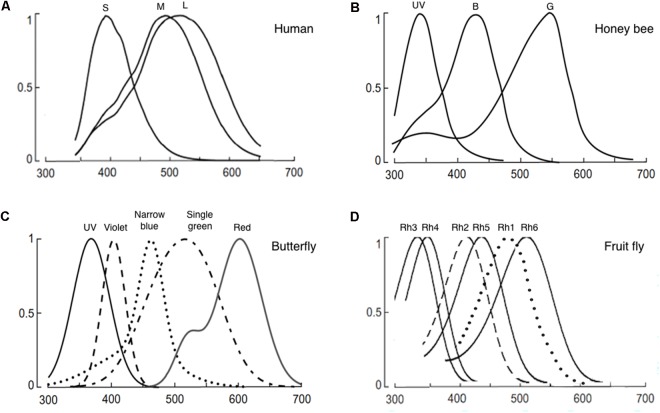
Normalized photoreceptor spectral sensitivities of: **(A)** Human, **(B)** honeybee (*Apis mellifera*), **(C)** butterfly (*Papilio xuthus*), and **(D)** fruit fly (*Drosophila melanogaster*). Panels **(A–C)** are reused with permission from the publication (Osorio and Vorobyev, 2008). Panel **(D)** is modified from (Salcedo et al., 1999). In panel **(C)**, only the spectral specificity of narrowband receptors is shown among the known eight photoreceptors of the butterfly.

Color vision is the internal representation of spectral distribution in the environment. It involves detection of multiple chromatic inputs in the peripheral sensory organ and multi-step extraction and integration of the chromatic information in the brain. For this reason, even under identical conditions, animals that have different photoreceptors, the detector of chromatic signals, and differently wired brains show different perception in chromaticity (**Figure 1**). In this review, we first examine insect photoreceptors and the relationship between animals’ color vision and ecology. Second, we review the visual circuits that process chromatic information. We discuss the electrophysiological studies that reveal color coding in bees and the genetic studies that identify neuronal correlates of color vision and innate spectral preference in fruit fly. Third, we discuss insect behaviors involving “true color vision” and “wavelength-specific behavior,” specifically in bees, butterflies, and flies. We include examples that demonstrate higher color vision, such as color contrast and constancy.

## Photoreceptors

Color vision starts with light detection in the peripheral sensory organ, the eyes. Insects have distinctive compound eyes that consist of repetitive visual units called ommatidia. Each ommatidium houses eight to nine photoreceptors known as retinula cells. Photoreceptors have rhabdomeres, microvillar structures where visual pigments are densely packed. Individual visual pigment consists of an opsin protein covalently linked to light-sensitive retinal-based chromophore. Upon illumination, photon absorption by visual pigment trigger a series of phototransduction cascade, starting from isomerization of its chromophore and subsequent conformational change of the linked opsin protein (Chittka and Menzel, 1992; Montell, 1999).

Internal color representation of the external world is determined by spectral sensitivity, diversity, and spatial distribution of photoreceptors as well as screening pigments around the rhabdoms. Spectral sensitivity is the probability that a photon of a particular wavelength is detected by the photoreceptors. Spectral sensitivity of various insect eyes has been established by intracellular recording, electroretinogram, and spectrophotometry (Menzel and Blakers, 1976; Bertrand et al., 1979; Peitsch et al., 1992; Salcedo et al., 1999; Cronin et al., 2000; Skorupski and Chittka, 2010; Chen et al., 2013; Huang et al., 2014). Most insects have UV, blue, and green receptors whose spectral specificity peaks around 350, 440, and 530 nm (**Figure 1**; Briscoe, 2000; Briscoe and Chittka, 2001). However, the number of different types of photoreceptors and the resultant spectral range covered by the receptors differ widely among species (Briscoe and Chittka, 2001; Kelber et al., 2003). For example, the cockroach *Periplaneta americana* have only two types of photoreceptors that sense UV and green. In contrast, the butterfly *Papilio xuthus* have five types of receptors that sense UV, blue, violet, green, and red (**Figure 1**). The dragonfly *Sympetrum rubicundulum* and red housefly *Musca domestica* also have five types of receptors. However, variation in spectral specificity was found among *P. xuthus*, *S. rubicundulum*, and *M. domestica* (Kelber et al., 2003). The bluebottle butterfly *Graphium sarpedon nipponum* has 15 types of photoreceptors (Chen et al., 2016). Recent analysis of RNA transcripts from the eyes of 12 dragonfly species also discovered as many as 11–30 visual opsin genes (Futahashi et al., 2015). Based on information theory, Barlow (1982) argued that trichromacy provides separate dimensions of hue, saturation, and brightness and is sufficient to support coding of most variation in natural spectra in the human visible spectrum (400–700 nm). A substantial number of Odonata and Lepidoptera insects have four types of photoreceptors, with a red-photoreceptor with a spectral sensitivity around 600 nm and UV-, green-, and blue-photoreceptors (Peitsch et al., 1992). Among these insects, it was shown that yellow swallowtail butterfly, *P. xuthus*, have tetrachromacy (Koshitaka et al., 2008). The higher dimension color space of tetrachromacy covers a broader spectral range (from UV to red) and could potentially optimize color constancy (Kelber et al., 2003). Increasing the dimension of color space beyond tetrachromacy, however, has only marginal effect in color vision. Then, why do many insects have more than four types of photoreceptors in their eyes including the extreme case of the bluebottle butterfly *G. sarpedon nipponum* with 15 types of photoreceptors? First, it should be noted that the total number of photoreceptor types or opsin genes does not equate to the dimension of color space. Only the photoreceptor types that pipeline into the downstream neural circuit for opponency-based chromatic comparisons contribute to the dimensionality of the color space (see section “Chromatic Processing”). It is thus conceivable that some of the photoreceptors have evolved to mediate wavelength-specific or task-specific behaviors which are hard-wired behavioral responses triggered by a particular wavelength band. Such behaviors are highly dependent on intensity within each wavelength (Marshall and Arikawa, 2014). In this scenario, photoreceptors that mediate “wavelength-specific behavior” would be wired differently from the photoreceptors that mediate “true color vision.”

Lastly, spatial distribution of photoreceptors also affects color vision. Ommatidial heterogeneity in spectral sensitivity was observed in various insects that do not share ommatidial organization (Wernet et al., 2015). Ommatidial heterogeneity does not solely attribute to spontaneous nature of photoreceptor development since the insect retina is sometimes divided into large territories with different morphological or functional properties (Wernet et al., 2015). The ommatidial heterogeneity could shape color vision by affecting spatial representation globally across the eye or locally in particular regions that have been specifically adapted for certain tasks. The dorsal rim area, a band of few ommatidial rows along the dorsal head cuticle, detects the celestial polarization pattern for navigation. Interestingly, the R8 photoreceptors in DRA in *Drosophila melanogaster* express UV-sensing opsins instead of green- or blue-sensing opsins as in the R8s in the other part of the eye. Additionally, sexual dimorphic expression of green-absorbing visual pigments were found in the dorsal part of the eye of male *Apis mellifera*, which plays a critical function for their mating flights (van Praagh et al., 1980).

Evidence indicates that the spectral specificities of photoreceptors are well fitted to their biological need in their natural habitat (Montell, 1999; Hardie and Raghu, 2001; Hardie and Juusola, 2015). However, insects with very different life styles share similar or identical sets of colors. As discussed earlier, although spectral specificity of photoreceptors is a critical determinant of color vision, the cellular context where the photoreceptors are expressed could be equally important. This partly explains why some insects with very different life styles share similar or identical sets of photoreceptor types.

## Anatomy

**FIGURE 2:**
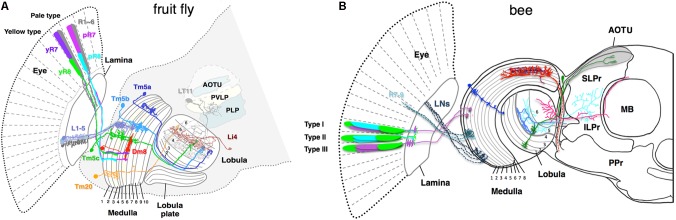
Schematic of color sensitive neurons in the optic lobe and the central brain **(A)** in fruit fly *Drosophila melanogaster*, **(B)** in bumblebee *Bombus impatiens*. **(A)** The fly ommatidia are classified into yellow-type (y) and pale-type (p) based on spectral specificity of the narrowband R7 and R8 receptors. The pR7, yR7, pR8, and yR8 express Rh3, Rh4, Rh5, and Rh6, respectively (see **Figure 1D** for their spectral specificity). L1–5 are lamina monopolar neurons, Tm5a-c and 20 are transmedullar neurons, Li4 is a lobula intrinsic neuron, LT11 is a lobula tangential neuron, AOTU (anterior optic tubercle protocerebrum), PVLP (posterior ventrolateral protocerebrum), and PLP (posterior lateral protocerebrum) are neuropil-dense regions in the protocerebrum in the fruit fly. **(B)** The axonal projections of bumblebee photoreceptors (R7–R9) and lamina neurons (LNs) to the medulla 1 and 2 layers are shown as dotted lines. Due to insufficient information for bumble bee, the ommatidial arrangement and innervation pattern of the photoreceptors of honeybee *Apis mellifera* are shown as reference (Menzel and Blakers, 1976; Kelber, 2016). Among the nine photoreceptors housed in each bee ommatidium, composition of the two distal photoreceptors vary in different types. The type I, II, and III contain one of each blue- and UV-receptors, two blue-receptors, and two UV-receptors, respectively. The medulla neurons marked in blue, red, and brown are transmedullar, large-field, and amacrine neurons, respectively. Neurons that are sensitive to color are anatomically segregated. Neurons in blue and green are examples that target only proximal lobula and both proximal and distal lobula, respectively. The downstream neurons that receive input from proximal lobula innervate anterior protocerebrum such as ILPr and SLPr. SLPr (superior lateral protocerebrum), ILPr (inferior lateral protocerebrum), PPr (posterior protocerebrum), and AOTU (anterior optic tubercle) are the neuropil-dense regions in the lateral protocerebrum in the bee. MB is mushroom body.

Visual information received by the compound eyes is transmitted to and processed by the optic lobes, which connect to the protocerebrum. The optic lobes consist of successive neuropils: the lamina, the medulla, and the lobula complex, which, in different insects, is either merged (in bees) or separated into the lobula and the lobula plate (in flies and butterflies) (**Figure 2**). The optic lobes contain regular columnar elements such as lamina cartridges, medullar and lobular columns, which correspond topographically to the facet pattern of the eyes and the visual field (Fischbach, 1979; Strausfeld and Okamura, 2007; Strausfeld et al., 2007; Takemura et al., 2008; Paulk et al., 2009a). Between successive neuropils, neural fibers cross over, forming the outer and inner optic chiasma so that neural presentation of the visual image is inverted in the medulla and then reverted in the lobula complex. In *Drosophila* and other higher *Dipterans*, the lamina receives inputs from only the broad-spectrum photoreceptors, R1–R6, thus being insufficient to mediate color vision. In the butterfly lamina, such as *P. xuthus*, the photoreceptors of different spectral classes form complex interconnections, which could potentially sharpen spectral sensitivity or form spectral opponency by mutual inhibition (Takemura et al., 2005).

Crossing the columnar units, the medulla and lobula complexes also have laminated structures. They are formed by afferents and dendritic arbors, which in turn elaborate in distinct strata to form synapses. In each stratum, extraction of different visual features occurs by integrating synaptic inputs from specific afferents terminating in particular strata. The *Drosophila* medulla is divided into 10 strata (M1–M10) where the M3 stratum receives both direct input from the narrow-spectrum photoreceptor R8 and indirect input from the broad-spectrum photoreceptors R1–R6 via L3. The M4–M6 strata also receive direct input from the other narrow-spectrum photoreceptor R7. The group of medulla neurons, including Tm5a/b/c and Tm20, that connect the strata 3, 5, and 8 to the deeper layers of the lobula (Lo4–6) are likely involved in chromatic information processing (Bausenwein and Fischbach, 1992; Melnattur et al., 2014).

Connectivity studies in *Drosophila* and electrophysiological studies in bees suggest that the deeper lobula layers (Lo4–6 in flies and Lo5 and 6 in bees) are involved in processing chromatic information (**Figure 2**; Bausenwein and Fischbach, 1992; Paulk et al., 2008; Melnattur et al., 2014; Otsuna et al., 2014; Lin et al., 2016). In *Drosophila*, the deeper lobula layers connect to the central brain via two classes of lobula neurons, the lobula columnar (LC) neurons and lobula tangential (LT) neurons (Otsuna and Ito, 2006; Wu et al., 2016). Specific types of the LC and LT neurons that receive inputs in deeper lobula layers target to distinct visual glomeruli in the ventrolateral protocerebrum (Otsuna and Ito, 2006; Wu et al., 2016), suggesting that these areas of the central brain might be involved in processing chromatic information. The pattern of chromatic responses of neurons in the retina, optic lobes, and central brain as well as their wiring pattern suggests that chromatic processing starts in the retina and continues in the medulla, the lobula, and the central brain (Matic, 1983; Srinivasan and Lehrer, 1985; Paulk et al., 2008, 2009a,b; Chen et al., 2013).

## Color-Driven Behavior

Color vision is a loose term that includes both “true color vision” and “wavelength-specific behavior.” “True color vision” is an ability to sense chromatic contrast independent of overall intensity. “True color vision” in non-human animals was demonstrated by showing that animals discriminate among chromatic contrasts independent of effective intensity contrasts after being trained for particular chromatic stimulus. On the other hand, more primitive “wavelength-specific behaviors,” such as phototaxis, are hard-wired behavioral responses triggered by a particular wavelength band and such behaviors are highly dependent on intensity within each wavelength. For example, the water flea, *Daphnia*, is attracted by green light which signals for algae but avoids damaging UV light near the surface (Lythgoe and Partridge, 1989).

About 100 years ago, von Krisch first demonstrated “true color vision” in insects by showing that honeybees can be trained to identify colors among different shades of gray, one of which matches the brightness of a target color. He trained the bees to land on the training color by pairing only the color with a positive cue among multiple colors that were simultaneously given as a choice. By taking the same approaches with different sets of training and test colors, later studies showed that honeybees have trichromacy like humans and can discriminate colors of different hues and saturation regardless of brightness (Werner et al., 1988; Spaethe et al., 2014).

The following “true color vision” phenomena have been extensively studied and verified in honeybees, blowflies, butterflies, and moths: color constancy, simultaneous contrast, and successive color contrast (Neumeyer, 1981; Fukushi, 1989). Color constancy is the phenomenon whereby an object’s apparent color remains constant despite changes in the hue changes in the illuminating light. For instance, two groups of a nocturnal hawkmoth *Deilephila elpenor* that were trained either to green or to turquoise in white light reliably identified the training color in yellow illumination despite that turquoise in yellow light generates almost the same quantum catch in the photoreceptor classes of the moth as green in white light (Kelber et al., 2002). Given that the color of illumination alters spectral composition of the reflected light from an object and the perceived hue of the object subsequently, color constancy is essential for reliable detection of biologically relevant objects under the variation in spectral distribution of natural daylight. How does color constancy work in insects? It was proposed that the spectral content that is common in the target object and its surroundings is subtracted through either desensitization of visual pigments that detect the common hue and/or by lateral inhibition between neighboring photoreceptor cells that express particular visual pigments (Neumeyer, 1981).

Next, simultaneous color contrast and successive color contrast are phenomena in which the hue of the target is altered by the color of its surrounding background and by pre-adaptation to a color prior to the test, respectively. Simultaneous chromatic contrast was well demonstrated in the choice experiment in *P. xuthus* (Kinoshita and Arikawa, 2014) in which the butterflies were trained to choose pale-green or orange among four other similar shades of colors that were presented as a color disk surrounded by a gray square background. When the background was changed to blue and to yellow, the butterflies that were trained to pale-green preferred blue-green and spring-green, respectively. When the background color was changed to green and to violet, the butterflies that were trained to orange preferred dark-orange and yellow-orange, respectively. It is intriguing that the degree of hue shift in the chosen colors matched the distance between the two background colors. This implies, for instance, that blue-green in the blue background would look similar to pale-green in the gray background. Consistently, it was also shown that a change in background color and pre-adaptation to a color prior to testing induced a change in hue shift in the color choice in honeybees (Neumeyer, 1980, 1981). Then, what would be the physiological basis of the observed color contrast? The direction of hue shift induced by the colored surrounds was the same as had been predicted on the basis of a selective sensitivity reduction (Neumeyer, 1980). This supports the possibility that it is due to a selective sensitivity reduction of photoreceptor cells involving changes in concentration of visual pigments in the photoreceptor cells or excitability of the photoreceptor cells (Neumeyer, 1981). Given that the strength of color contrast matched to the distance between the background colors, there could be a shared mechanism between color constancy and color contrast.

In addition to “true color vision,” many animals, including insects, also show “wavelength-specific behavior.” These include phototaxis toward specific wavelength of light and wavelength-specific behavior directed toward objects (Menzel, 1979; Kelber and Osorio, 2010). In contrast to “true color vision,” such behavior is highly dependent on intensity. The wavelength-specific behaviors are thought to be mediated by simple neural connections rather than complex chromatic comparisons (Marshall and Arikawa, 2014). In butterflies, which have up to twelve types of photoreceptors, it is likely that some selected chromatic channels are used for wavelength-specific tasks on top of their trichromatic or tetrachromatic color vision. The white butterfly *Pieris rapae* exhibits several wavelength-specific behaviors including attraction to UV/violet, reflex proboscis extension behavior to blue and egg laying to green (Hepburn, 1971; Davies and Gilbert, 1985). The innate wavelength-specific behaviors appear to be mediated by few channels directly without complex channel comparison.

Behavioral studies, in combination with molecular genetics, have been used to identify neural substrates for color vision. Diverse behaviors are regulated by color vision that may involve distinct sets of color-sensitive neurons. The combined behavioral and molecular genetic approach complements the anatomical and electrophysiological approach in that it enables identification of color-sensitive neural pathways that mediate particular behaviors. The fruit fly *D. melanogaster* is genetically tractable and ample toolkits for genetic manipulation are available (del Valle Rodriguez et al., 2012; Mohr et al., 2014). By taking the combinatorial approach in *Drosophila*, two studies identified neural pathways that mediate color vision. By testing whether selective restoration of photo-transduction in single or multiple types of photoreceptors restores “color vision” in blind mutant flies, one study demonstrated that broadband outer photoreceptors R1–R6 and one other narrowband photoreceptor R7 are sufficient to mediate “true color vision” (Schnaitmann et al., 2013). Contribution of the R1–R6 photoreceptor pathway to color vision was further supported by the necessity of lamina monopolar neurons, the neurons that are postsynaptic to R1–R6, in the color vision-mediated behavior. The other study reported that, four different types of transmedullar neurons, Tm5a/b/c and Tm20, act redundantly to mediate “true color vision.” These transmedullar neurons receive synaptic inputs from either or both narrowband inner photoreceptors R7 and R8 (Gao et al., 2008; Karuppudurai et al., 2014). When the chemical neurotransmission from different subsets of these neurons was blocked, color vision stayed intact in all cases except when outputs from all four types were inactivated collectively, suggesting that these neurons mediate color vision redundantly (Melnattur et al., 2014).

## CHROMATIC PROCESSING

How is chromatic information received by photoreceptors processed and color represented in the insect brains? Without additional mechanisms such as selective filtering (Mäthger et al., 2009) and chromatic blurring (Stubbs and Stubbs, 2016), a single photoreceptor type cannot discriminate chromatic content from intensity of light. Thus, color processing requires comparison of inputs between photoreceptors of different spectral specificity. Hering (1964) proposed that the inputs from different photoreceptor cells converge onto cells where they are combined in a spectrally antagonistic fashion so that the recipient cell is excited when the eye is stimulated by some wavelengths of light and is inhibited by stimulation from other wavelengths (Backhaus, 1991). The chromatic antagonism-based color processing is strongly supported by patterns of chromatic responses of neurons in multiple species including insects. In addition, observations from “true color vision” such as color constancy and simultaneous and subsequent color contrast consistently predict that cells that receive antagonistic cone inputs are spatially tuned. In other words, the chromatic neural circuits should project not only spectral reflectance of colored surfaces but also spatial layout of the scenes. In fact, color-responsive neurons in layer 2/3 of the primate V1 cortex were mostly sensitive to spatial patterns (Johnson et al., 2001; Gegenfurtner and Kiper, 2003).

Among insects, bees have been the chief subject of electrophysiological investigation of chromatic information processing (Menzel and Blakers, 1976; Hertel, 1980; Riehle, 1981; Menzel, 1985; Hertel and Maronde, 1987; Yang et al., 2004; Paulk et al., 2008, 2009a,b). According to their response patterns to colors, neurons in bee optic lobes and central brain are categorized into the three classes: broadband, narrowband, and color-opponent neurons (Hertel, 1980; Paulk et al., 2008, 2009a). Broadband neurons show little or no spectral specificity, showing the same direction of response to the range of light spectrum that covers two or more types of photoreceptor cells. In contrast, narrowband and color opponent neurons respond in a spectrally specific manner and thus are likely to be involved in chromatic processing. Narrowband neurons show high sensitivity only to a small portion of spectral sensitivity of a single photoreceptor type. The color opponent neurons, on the other hand, show excitatory responses to a particular wavelength and inhibitory responses to other wavelengths or vice versa. When the two wavelengths are presented together, the excitatory response of color-opponent neurons is partially or completely suppressed (Paulk et al., 2009a). The narrowband and color-opponent responses, together with the observation that the maximum sensitivity of some narrowband neurons lies between the sensitivity maximum of different photoreceptor cells (Hertel, 1980), strongly support Hering’s chromatic-antagonism theory in bees. Surprisingly, chromatic and spatial antagonism was frequently found to be uncoupled in bees (Kien and Menzel, 1977) and so far, no double-opponent cells have been found in insects. Although this issue needs to be revisited with more systematic probing of neural activity of color-responsive neurons, the observations suggest a possible difference in the strategy of color coding, especially the color constancy mechanism between insects and vertebrates (Hertel, 1980).

Where in the color circuit is the information from different photoreceptor types compared to generate color opponent signal? Ultrastructural studies on the fly medulla and the butterfly lamina showed that the terminals of different photoreceptor types form mutual synapses, which could potentially shape spectral sensitivity or form color opponency by mutual inhibition (Gao et al., 2008; Takemura et al., 2008). Recent study in *Drosophila* reported that opponency-based color processing indeed occurs at the photoreceptor level (Schnaitmann et al., 2018). Each ommatidium in the fly eye houses a pair of R7 and R8 narrowband photoreceptors. Based on spectral specificity, each ommatidium is classified into either pale (p) or yellow (y) type: the pale-type ommatidia house short UV-sensitive pR7 and blue-sensitive pR8 while the yellow-type ommatidia house long-UV-sensitive yR7 and green-sensitive yR8 (**Figure 1**). Interestingly, R7 and R8 photoreceptors from the same ommatidium form inhibitory synaptic connections with each other and encode short-UV/blue (for pale type) or long-UV/green (yellow type) color opponent signals. The histamine receptor HisCl1 mediates direct mutual inhibition between R7s and R8s while the second histamine receptor Ort is required for yet uncharacterized feedback inhibition and R8s’ color opponency (Schnaitmann et al., 2018). As spectrally antagonistic processing already occurs at the R7/R8 photoreceptor terminals, the downstream neurons, Tm20 and Tm5a/b/c, which have been implicated in color vision (Melnattur et al., 2014), receive color-opponent inputs. In addition to R7 and R8, Tm5a/b/c and Tm20 also receive indirect inputs from the broad-spectrum R1–R6 via the laminal neurons L3 (Gao et al., 2008; Karuppudurai et al., 2014). Whether these neurons further process color information, such as incorporating the broad-spectrum information, is currently unknown.

To get further insights into mechanisms underlying chromatic processing, color-responsive neurons in the optic lobes and protocerebrum of the bumble bee *Bombus impatiens* were systematically investigated by electrophysiology and then correlated with their arborization patterns (Paulk et al., 2008, 2009a,b). Based on the breadth and location of their primary dendritic branches as well as their axonal targets, the medulla neurons are classified into three types: large-field, amacrine, and transmedullary neurons (**Figure 2B**). Intracellular recording revealed that all three types show broadband, narrowband, and color-opponent responses although the fraction that showed narrowband and color-opponent responses varied in different groups (Paulk et al., 2009a). The bumblebee medulla is subdivided into eight layers with layers 1 and 2 receiving primary color inputs from photoreceptors and lamina neurons (**Figure 2B**). Interestingly, different classes of color-sensitive neurons are segregated in distinct medulla layers: neurons arborizing in layers 1–3 are mostly broadband or narrowband while most color-opponent neurons are the large-field and amacrine neurons with arborizations in the layers 4 and 5. This suggests multi-stage color processing in the medulla with color opponency enabling primarily in the proximal medulla (layers 4 and 5). Furthermore, color information could be encoded in the temporal response patterns. Depending on the baseline spike rate, direction and timing of change in spike rate, the temporal responses of color-sensitive neurons could be categorized into phasic-tonic excitatory, phasic excitatory, on-off excitatory, and tonic inhibition-off responses (Paulk et al., 2009a). The response patterns of large-field and amacrine medulla neurons are predominantly phasic-tonic excitatory while the transmedullary neurons mostly showed tonic inhibition-off responses. Finally, color-sensitive neurons in the medulla innervate the protocerebrum either directly or indirectly via lobula neurons, suggesting parallel color information streams.

Anatomical segregation of color-sensitive neurons was more pronounced in the lobula and the protocerebrum. Color opponent and narrowband responses were observed in the neurons that extend dendrites in the proximal lobula (layers 5 and 6) while the neurons that receive input from the distal lobula (layers 1–4) were mainly motion-sensitive. Many neurons in the layers 5 and 6 project to the dorsal and lateral protocerebrum as well as mushroom body calyces (**Figure 2B**). The protocerebrum is a part of central brain that receives input from both the medulla and lobula. The lateral protocerebrum is sub-divided into five neuropil-dense regions of the superior lateral protocerebrum (SLPr), the inferior lateral protocerebrum (ILPr), the posterior protocerebrum (PPr), the lateral horn, and the anterior optic tubercle (AOTU). Most of tangential lobula neurons in the proximal lobula projects into the SLPr or ILPr in the anterior lateral protocerebrum whereas distal lobula neurons mostly targets PPr. The fact that anterior lateral protocerebrum were enriched with either narrowband or color-opponent neurons and that such neurons were barely seen in either medial protocerebrum or PPr suggest that color-sensitive and motion-sensitive neurons are segregated in the lateral protocerebrum along the anterior–posterior axis. A recent calcium imaging study in honey bees showed distinct regions of AOTU are activated by different colors of light, suggesting a spatial segregation of color processing in AOTU (Mota et al., 2013). The color-sensitive medulla, lobula, and anterior protocerebrum neurons showed habituation upon repeated stimulation with same colors. Notably, such habituation was reversed at least in proximal lobula neurons when the color of the stimulus was changed (Paulk et al., 2008).

Honeybees also exhibit strong positive phototactic behaviors. All three types of photoreceptors, blue, green, and UV, contribute to phototaxis without significant deviation from simple linear summation of all channels (Menzel and Greggers, 1985). The lamina monopolar cell M1, which pools all receptor inputs from a single ommatidium, or a functionally equivalent type in deeper optic lobes might be involved in the phototactic behavior (Menzel, 1974; de Souza et al., 1992). Notably, the whitefly *Trialeurodes vaporariorum* exhibits positive phototaxis toward yellow light but negative phototaxis toward blue light, thus showing clear spectral selectivity. However, their phototactic responses also reflect a simple summation of spectral components (Coombe, 1981). Simple spectral additivity strongly suggests that true color vision is not involved in the phototactive behaviors of these two species.

In *Drosophila*, where all receptor types contribute to phototaxis, significant non-additive effects were found in spectral preference assay and color mixing experiments, suggesting antagonistic interactions among the broad-spectrum R1–R6 receptors and the narrow spectrum receptors R7 and R8 (Fischbach, 1979; Gao et al., 2008; Yamaguchi et al., 2010). Quantitative spectral preference assay revealed that flies prefer UV to green light by about two orders of magnitude (Gao et al., 2008). Targeted manipulation of neuronal activity further showed that the UV preference depends on the UV-sensing R7 photoreceptors and its downstream neurons, the amacrine neuron Dm8 and the transmedulla neuron Tm5c. Circuit mapping by serial EM reconstruction and the GRASP method further revealed that Dm8 neurons receive 13–16 R7 photoreceptor inputs and provide output for the projection neuron Tm5c (Melnattur et al., 2014). Notably, Tm5c receives inputs from R8 and Dm8 and has been implicated in color vision, suggesting a partial overlapping between the spectral preference circuit and color vision circuit. While the deeper lobula has been implicated in chromatic processing, the lobula neurons and circuits involved in wavelength-selective behaviors are not well understood. Using simple phototaxis assays, it was found that inactivating the large LT neuron LT11 caused reduced phototaxis toward 440 nm wavelength band (Otsuna et al., 2014). LT11 receives inputs from all four chromatic Tm neurons, Tm5a/b/c and Tm20, across the entire hemi-visual field of the lobula and projects an axon to optic glomeruli in the posterior ventrolateral protocerebrum. The connectivity of LT11 and other putative chromatic lobula neurons suggests that the chromatic information processing in the lobula involves both spatial and chromatic integration (Lin et al., 2016).

## Outlook

Here we review studies that advanced our mechanistic understanding of color vision and wavelength selectivity in insects. Emerging studies in behaviors, anatomy, and physiology in different insect species demonstrated that insects, like vertebrates, utilize opponency-based processing for “true color vision” despite their differences in ecology and visual system structures. As in the vertebrate retina, color-opponent processing occurs at the level of the first synapse in *Drosophila* but with a very different cellular and molecular implementation from that of vertebrates. Chromatic information is extracted from photoreceptors through proximal to distal optic lobes sequentially and appears to be represented as narrow spectral and color-opponent signals. While the synaptic mechanism underlying color opponency and narrow spectral sensitivity is being uncovered, the mechanisms for spatial integration of color information and color constancy, which likely occur in the optic lobe circuit, are entirely unknown. Answers to these key questions in color vision would require a truly multi-disciplinary approach to determine the spectral sensitivity, connectivity, and behavioral roles of identified color neurons in the chromatic pathways within one single species.

On the other hand, the mechanisms for wavelength selectivity appear to differ among different insect species and likely reflect their distinct ecological needs. *Drosophila* compares photoreceptor channels and their wavelength selective pathway partially intermixes with color-vision pathways. Butterflies, with their added-extra sets of photoreceptors, appear to utilize a few channels directly without complex comparison. The study of wavelength selectivity thus provides a unique opportunity to understand the neural mechanism coupling chromatic stimuli to species-specific behaviors.

## Author Contributions

All authors listed have made a substantial, direct and intellectual contribution to the work, and approved it for publication.

## Conflict of Interest Statement

The authors declare that the research was conducted in the absence of any commercial or financial relationships that could be construed as a potential conflict of interest.
